# The Modern Treatment of Diphtheria

**Published:** 1894-08

**Authors:** 


THE MODERN TREATMENT OF DIPHTHERIA.—Fischer, of New York, contributes the following conclusions to the *Post Graduate* for April, 1894:

1. Local disinfection,—careful, distinct nasal antisepsis by means of naso-pharyngeal irrigation; this can be done by irrigating with warm salt water (1 drachm to the pint); the addition of bichloride of mercury (1 grain to the pint), or of borax (1 to 2 drachms), or acid salicylic (4 grains to the pint), is regarded as an improvement by some. It should be thrown with enough force to flow out of the other nostril and partly by the throat. Tearing the membrane or using force will cause epistaxis, and is not advisable. Jacobi says in his treatise of diphtheria (page 208): "When they see benzoate of sodium destroying bacteria in a glass vessel, they take it for granted that the human system will permit of the same action as a glass vessel. Thus benzoate of sodium is sent into the stomach or pulmonary artery under the order to do the same as it does in the laboratory. The drug has, in consequence, had a short life, after having been extolled in a very limited time by microscopists, etc."

Jacobi concludes by saying that as an antidiphtheritic or antipyretic it utterly failed. Frequent applications of pepsin, trypsin or papayotin with a medicine dropper may be indicated in some abstract cases. Naso-pharyngeal irrigation will cause the neighboring enlarged glands to become reduced in size quicker than all other treatment combined.

2. Sponging with peroxide of hydrogen hourly.

3. Internal administration of stimulant drugs,—ferric chloride for tonic effect. Alcohol, no matter at what stage, is indicated in all cases of septicemia, be they diphtheritic or not.

4. Liquid diet,—milk, beef-tea, milk-punch; rectal feeding if necessary.

5. Local applications of cotton swabs moistened with bichloride (1 to 1000 to 1 to 500), applied with great pressure every two hours, as an abortive plan of treatment, if the case is seen early.

6. Antipyretics.—If at all useful, salicylate of sodium is the best, in doses of 2 to 15 grains every two hours or more, according to the age and tolerance of the patient. If the drug is not well borne, the antipyrin or antifebrin may be given.

The temperature in uncomplicated cases of diphtheria has given little trouble, except when complicated by scarlatina. Cold applications—cracked ice and ice cream—are very grateful to patients.

Calomel, to empty the bowels and reduce the temperature, is a mercurial salt, besides being a valuable intestinal antiseptic. Orange and lemon juice are also highly beneficial. The complications and their management must be considered in each case. So must the question of intubation and tracheotomy, to which the author only refers. Every case must be studied, and the treatment based on the previous habits, the general condition, the severity of the attack and the amount of the infection.—*Fort Wayne Medical Magazine.*

